# Diagnosis, treatment, and consequences of anastomotic leakage in colorectal surgery

**DOI:** 10.1007/s00384-016-2744-x

**Published:** 2017-01-09

**Authors:** Bodil Gessler, Olle Eriksson, Eva Angenete

**Affiliations:** Department of Surgery, Scandinavian Surgical Outcomes Research Group (SSORG), Institute of Clinical Sciences, Sahlgrenska Academy, University of Gothenburg, Sahlgrenska University Hospital/Östra, SE-416 85 Gothenburg, Sweden

**Keywords:** Colrectal surgery, Anastomotic leakage, Postoperative complications

## Abstract

**Purpose:**

The aim of this study was to explore the choice of modality for diagnosis, treatments, and consequences of anastomotic leakage.

**Methods:**

This is a retrospective study of consecutive patients who underwent surgery that included a colorectal anastomosis due to colorectal cancer, diverticulitis, inflammatory bowel disease (IBD), or benign polyps.

**Results:**

A total of 600 patients were included during 2010–2012, and 60 (10%) had an anastomotic leakage. It took in mean 8.8 days (range 2–42) until the anastomotic leakage was diagnosed. A total of 44/60 of the patients with a leakage had a CT scan of the abdomen; 11 (25%) were initially negative for anastomotic leakage. Among all leakages, the anastomosis was taken down in 45 patients (76.3%). All patients with a grade B leakage (*n* = 6) were treated with antibiotics, and two also received transanal drainage. The overall complication rate was also significantly higher in those with leakage (93.3 vs. 28.5%, *p* < 0.001), and it was more common with more than three complications (70 vs. 1.5%, *p* < 0.001). There was a higher mortality in the leakage group.

**Conclusion:**

This study demonstrated that one fourth of the CT scans that were executed were initially negative for leakage. Most patients with a grade C leakage will not have an intact anastomosis. An anastomotic leakage leads to significantly more severe postoperative complications, higher rate of reoperations, and higher mortality. An earlier relaparotomy instead of a CT scan and improved postoperative surveillance could possibly reduce the consequences of the anastomotic leakage.

## Introduction

Anastomotic leakage remains a severe complication after abdominal surgery with considerable morbidity and mortality [1–11]. The frequency ranges from 1.8 to 19.2% and depends partly on different risk factors [4, 12–20]. Risk factors for leakage have been extensively studied, and the most frequent factors mentioned are male sex, high age, a low anastomosis, malignant disease, high American Society of Anesthesiologists (ASA) score, long operation time, emergency operation, preoperative radiotherapy, and perioperative blood loss or transfusion [4, 13, 18, 21–26]. There is no universal grading of the leakages, but the definition proposed by Rahbari et al. is often used for rectal cancer and consists of a three-grade scale. Grade A requires no therapeutic intervention; grade B includes active intervention without laparotomy, and if laparotomy is required, the leakage is classified as grade C [27]. The diagnostic methods commonly used when a leakage is suspected are CT scan, contrast enema, endoscopic examination, and reoperation [28]. The leakage may be diagnosed at different time points postoperatively, and there are theories that early and late leakages are different entities. One suggestion is that a later diagnosed leakage only has more subtle symptoms, and thus, more is accurately described as discrete than late [29–33]. Treatment of an anastomotic leakage differs with the severity and the location of the anastomosis. Often, there is a high frequency of permanent stoma after a reoperation and anastomotic take down. Salvage of the anastomosis is more common in grade A and B leakages with the treatment consisting of drainage and/or antibiotics [3, 34–36]. Despite the increased knowledge of an anastomotic leakage, there is still a need for studies in an unselected cohort of patients receiving surgery for both benign and malignant diseases, to try to improve results after the anastomotic leakage has occurred.

The aim of this study was to explore the choice of modality for diagnosis, treatments, and consequences of anastomotic leakage in colorectal surgery in an unselected population.

## Methods

### Study design

This is a retrospective study of a consecutive series of patients, over 16 years old, between the first of January 2010 to the 30 June 2012, who underwent colorectal surgery that included an anastomosis due to colorectal cancer, diverticulitis, inflammatory bowel disease (IBD), or benign polyps. All patients were treated at the Sahlgrenska University Hospital/Östra in Sweden, that serves approximately 700,000 inhabitants. The Nordic Medico-Statistical Committee (NOMESCO) Classification of Surgical Procedures version 1.9 was used to identify all patients. End follow-up was set to 6 May 2014 or the date of death. The median time of follow-up was 32 months (interquartile range (IQR) = 16).

### Included variables

The medical records were studied, and data was collected including patient-related information such as demography (date of birth, sex, weight, height) and ASA classification. The following comorbidity was registered: diabetes mellitus, hypertension, other cardiovascular disease (heart failure, heart attack, angina pectoris, or heart valve diseases), neurologic disease (stroke, epilepsy), and chronic obstructive pulmonary disease (COPD)/asthma. The diagnoses were identified using the International Statistical Classifications of Diseases and Related Health Problems 10 (ICD-10 codes). In addition to medical records, information was extracted from a health declaration that the patients filled in prior to surgery. Perioperative and postoperative variables including timing of surgery, type of operation, blood loss, hospital stay, complications (using the Clavien-Dindo classification system [37]), reoperations, and mortality were included in the database.

### Exclusion criteria

Exclusion criteria were resection without an anastomosis to the colon or rectum, when the surgery was considered a reoperation, reversal of a stoma, ileo pouch-anal anastomosis, and patients who moved shortly after the procedure (thus, no follow-up was possible) and when records had missing data from the surgery.

### Definitions

Anastomotic leakage was defined as any clinical signs of leakage, confirmed by radiological examination, endoscopy, clinical examination of the anastomosis (i.e., palpation of the anastomosis), or reoperation. The leakages were graded retrospectively according to the system proposed by Rahbari et al. [27]. Anastomosis takedown was defined as an interruption of the continuity of the bowel and the formation of a stoma. The blood loss was the volume noted by the anesthetic nurse during surgery. The surgical approach was divided into three groups: laparoscopy, laparotomy, and conversions from laparoscopy to open surgery, but in statistical calculations, the converted group is in the laparoscopic group as intention to treat. Anastomosis not taken down, salvage was defined as preservation of the bowel continuity with repair of the anastomosis or conservative treatment with or without drainage or antibiotics. Death was recorded within 30 and 90 days from index surgery. Time to diagnosis of a leakage was calculated as days between the index operation and diagnosis of the leakage with reoperation or CT abdomen or CT rectal contrast or with endoscopy or when fecal containing fluid was seen in the drainages. Total hospital stay included a second admission to hospital if the cause was anastomotic leakage or complications thereof. A stoma was counted as permanent if it was present at the end of follow-up time.

### Statistical analysis

The statistical calculations were performed using the IBM SPSS Statistics version 22.0. The study is mainly descriptive, and therefore, univariate statistical calculations were used. Chi-squared tests (categorical variables) or Mann-Whitney tests (continuous variables such as BMI or blood loss) were applied in comparison of groups. Fishers exact test was used if the number of categorical observations were fewer than five. Mean with range or median with interquartile range was used as descriptive statistics. Significance was defined as *p* value <0.05.

## Results

**Fig. 1:**
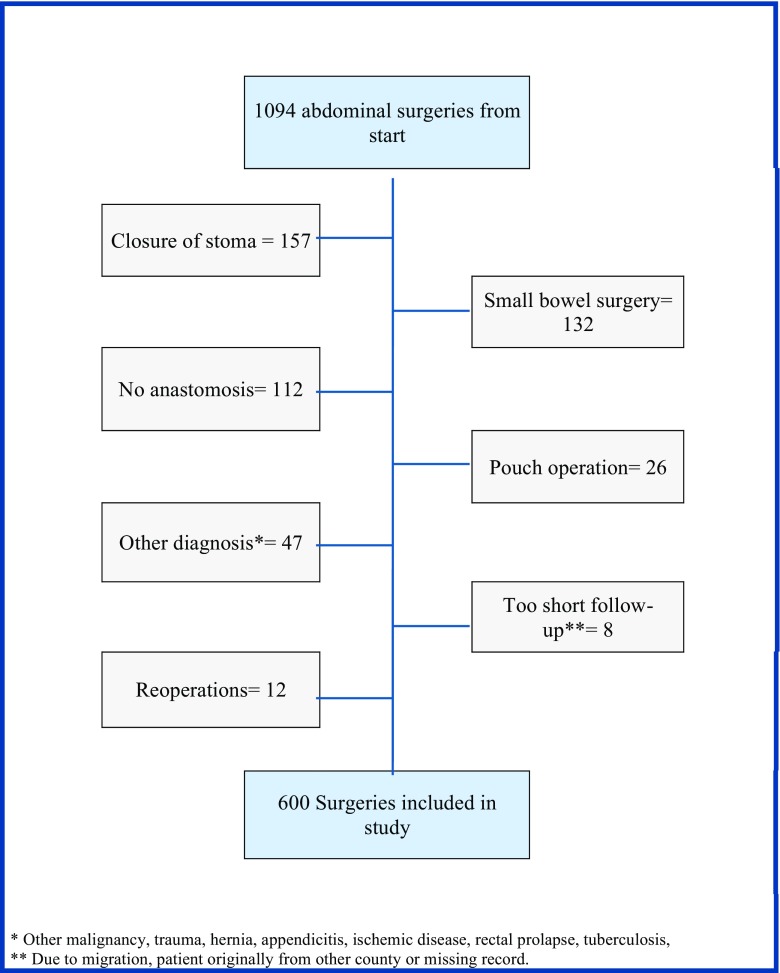
Flowchart of exclusion

A total of 1094 patients were identified; after exclusion, 600 consecutive patients who underwent a colorectal surgical procedure that included a primary anastomosis due to colorectal cancer, diverticulitis, IBD, or benign polyps were included (Fig. 1). Median age was 68.4 years (IQR 18), and there were slightly more women (50.8%) than men. Malignant disease was the reason for surgery in 487 (81.2%), and among these, 396 were colon cancer and 91 were rectal cancer. Sixty patients were found to have an anastomotic leakage resulting in an overall incidence of 10%. Anastomotic leakages were more common in rectal resections with a stapled anastomosis and when a defunctioning stoma was used, see Table 1 for details.

**Table 1:** Patient characteristics and anastomotic leakage

	Anastomotic leakage *n* = 60	No anastomotic leakage *n* = 540	Rate of anastomotic leakage	Comparison regarding anastomotic leakage	OR (95% CI)	*p* value
Age median (interquartile range)	67.3 (16)	68.6 (18)			−5.0, 2.4	0.485
Gender				Male/female	1.5 (0.9, 2.6)	0.134
Male	35	260	11.9%			
Female	25	280	8.2%			
ASA score (missing = 6)				ASA I–II/ASA III–IV	1.1 (0.6, 2.0)	0.747
ASA I–II	44	381	10.4%			
ASA III–IV	16	153	9.5%			
BMI (missing = 6)				BMI ≤25/>25	1.2 (0.7, 2.1)	0.495
≤25	32	260	11.0%			
>25	28	274	9.3%			
Diagnosis				Malignant/benign	1.4 (0.6, 2.8)	0.423
Malignant disease	51	436	10.5%			
Benign disease	9	104	8.0%			
Comorbidity^a^				Comorbidity yes/no	1.3 (0.7, 2.3)	0.349
0	22	232	8.7%			
1	31	250	11.0%			
≥2	7	58	10.8%			
Smoking (missing = 3)				Smoking yes/no	1.9 (0.9, 3.9)	0.066
Yes	11	56	16.4%			
No	49	481	9.2%			
Timing				Elective/emergency	1.4 (0.6, 3.4)	0.423
Elective	54	466	10.4%			
Emergency	6	74	7.5%			
Surgical approach				Laparoscopy and converted/open^b^	0.9 (0.5, 1.9)	0.836
Open	49	435	10.1%			
Laparoscopy	9	71	11.3%			
Conversion to Laparotomy	2	34	5.6%			
Surgical procedures						0.02
Right hemicolectomy	17	225	7.0%			
Left hemicolectomy	5	63	7.4%			
Sigmoid colectomy	11	117	8.6%			
Rectal resection	22	95	18.8%			
Total colectomy	2	14	12.5%			
Other colonic anastomosis^c^	3	26	10.3%			
Anastomosis technique (missing 7)				Stapled/hand-sewn	2.8 (1.5, 5.1)	0.001
Stapled	42	259	14.0%			
Hand-sewn	15	276	5.5%			
Stoma				Stoma/no stoma	2.8 (1.5, 5.2)	<0.001
Defunctioning stoma	18	71	20.2%			
No defunctioning stoma	42	469	8.2%			
Perioperative blood loss (missing 28)				>300/<300 ml	1.6 (0.9, 2.9)	0.097
>300 ml	37	274	11.9%			
<300 ml	21	254	7.6%			
Blood loss (ml) mean (range) (missing 14)	516 (0–2200)	426 (0–4000)				0.534

^a^Comorbidity: diabetes mellitus, hypertension, cardiovascular disease, cerebral disease, kidney disease, asthma/chronic obstructive pulmonary disease

^b^“Conversion to laparotomy” is counted in the laparoscopy group when comparison is made

^c^Other colonic anastomosis: transverse colectomy and other non-standard colectomies

### Diagnosis of anastomotic leakage

The time until diagnosis was in mean 8.8 days (range 2–42). CT scan was the most common diagnostic method with a total of 44/60. Of these, 11 (25%) were negative and 33 (75%) positive for anastomotic leakage. Although numerical differences indicating both shorter time to diagnosis (4.3 vs. 9.3 days) and shorter hospital stay (22 vs. 29.9 days) for patients diagnosed at surgery compared to all other diagnostic methods, this was not statistically significant (Table 2). A total of 12/60 (20%) patients were diagnosed with leakage after readmission.

**Table 2:** Diagnostic method of the anastomotic leakage

	Diagnosed at reoperation *n* = 7	Other diagnostic methods^a^ *n* = 53	OR (95% Cl)	*p* value
Days to leakage diagnosis, mean (range)	4.3 (2–9)	9.3 (2–42)		0.075
Days in hospital, mean (range)	22 (5–46)	29.9 (4–101)		0.321
30-day mortality *n* (%)	0	3 (6.4%)	1.3 (1.1, 1.5)	1.000
90-day mortality *n* (%)	0	5 (10.6%)	1.3 (1.1, 1.5)	0.575

^a^CT abdomen, CT with rectal contrast, endoscopic, or combination of more than one method

### Treatment of anastomotic leakage

**Fig. 2:**
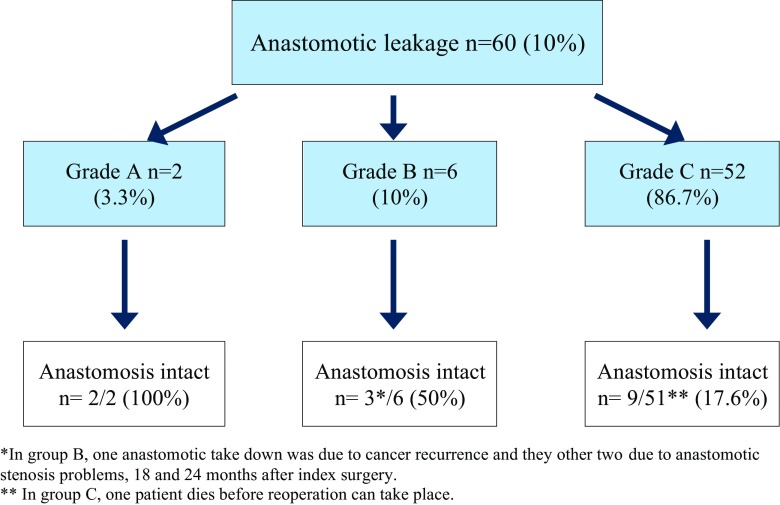
Flowchart over anastomotic leakages

One patient, with a grade C anastomotic leakage, died before surgery could take place. Among all leakages, grades A–C, the anastomosis was taken down in 45 patients (76.3%) and bowel continuity was intact in 14 patients (23.7%). Two patients in the grade B group later had their anastomosis taken down due to anastomotic stenosis and one due to local recurrence of cancer (Fig. 2). All patients in grade B group (*n* = 6) were treated with antibiotics; two also received transanal drainage.

### Postoperative complications and reoperations

**Fig. 3:**
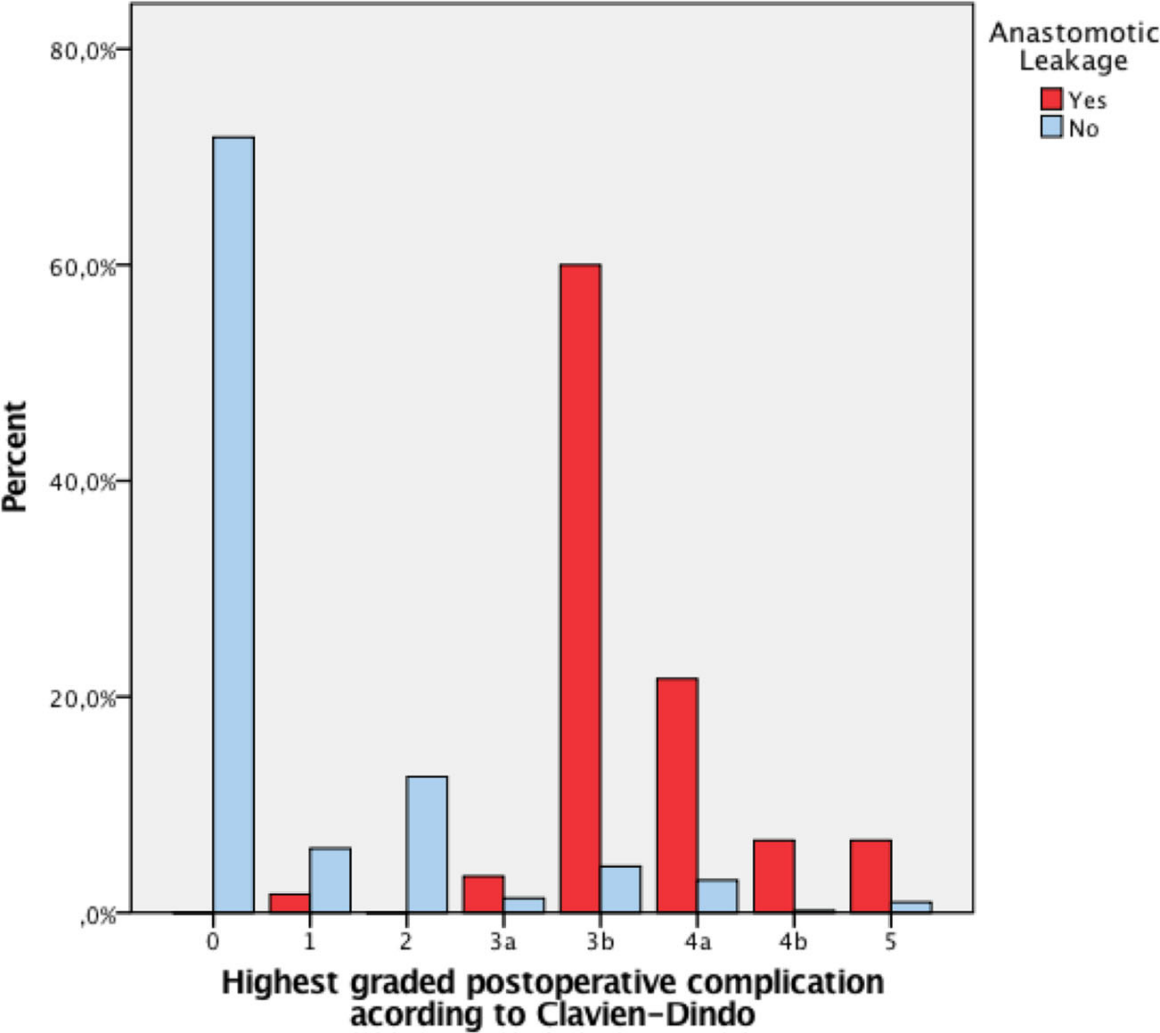
Postoperative complications—Clavien-Dindo

It was more common with a reoperation in patients with an anastomotic leakage compared to patients without leakage: 91.7% (*n* = 55) vs. 5.4% (*n* = 29) (*p* < 0.001). The overall complication rate was also significantly higher in those with leakage (93.3 vs. 28.5%, *p* < 0.001), and it was more common with more than three complications (70 vs. 1.5%, *p* < 0.001). However, the rate of wound infection and pneumonia did not differ between the groups; for details, see Table 3. More severe complications according to Clavien-Dindo were seen in patients with anastomotic leakage compared to patients without leakage (Fig. 3).

**Table 3:** Postoperative complications and morbidity

	Anastomotic leakage (*n* = 60)	No anastomotic leakage (*n* = 540)	OR (CI 95%)	*p* value
Days in hospital, mean (range)	29.0 (4–101)	9.4 (2–54)	14.5, 24.7	<0.001
30-day mortality *n* (%)	3 (5.0%)	3 (0.6%)	9.4 (1.9, 47.8)	0.015
90-day mortality *n* (%)	5 (8.3%)	11 (2.0%)	4.4 (1.5, 13.0)	0.004
Reoperation within 12 months *n* (%)	55 (91.7%)	29 (5.4%)	193.9 (72.1, 521.1)	<0.001
≥3 postoperative complications *n* (%)	42 (70.0%)	8 (1.5%)	155.2 (63.7, 377.9)	<0.001
Number of patients with one or more complications other than anastomotic leakage *n* (%)	56 (93.3%)	154 (28.5%)	35.1 (12.5, 98.4)	<0.001
Wound infection *n* (%)	5 (8.3%)	35 (6.5%)	1.3 (0.5, 3.5)	0.585
Wound dehiscence *n* (%)	5 (8.3%)	14 (2.6%)	3.4 (1.2, 9.8)	0.016
Abscess *n* (%)	27 (45%)	13 (2.4%)	33.2 (15.7, 70.2)	<0.001
Fistula *n* (%)	6 (10%)	4 (0.7%)	14.9 (4.1, 54.4)	<0.001
Bleeding *n* (%)	6 (10%)	18 (3.3%)	3.2 (1.2, 8.5)	0.012
Pneumonia *n* (%)	4 (6.7%)	12 (2.2%)	3.1 (1.0, 10.1)	0.066
Sepsis *n* (%)	5 (8.3%)	6 (1.1%)	8.1 (2.4, 27.4)	<0.001
Other infection^a^ *n* (%)	12 (20%)	34 (6.3%)	3.7 (1.8, 7.7)	<0.001
Cardiovascular complications^b^ *n* (%)	9 (15%)	18 (3.3%)	5.1 (2.2, 12.0)	<0.001
Respiratory complications *n* (%)	13 (21.7%)	4 (0.7%)	37.1 (11.6, 118.2)	<0.001
Renal failure *n* (%)	4 (6.7%)	2 (0.4%)	19.2 (3.4, 107.3)	0.001
Other complications^c^ *n* (%)	15 (25%)	29 (5.4%)	5.9 (3.0, 11.8)	<0.001

^a^Urinary infection, candida, or unknown source to infection

^b^Myocardial infarction, hypotension, heart failure, atrial fibrillation

^c^Severe pain, stoma-related complications, pancreatitis, peripheral neural damage, embolism, and intestinal obstruction

### Mortality

There was a significant increase in mortality among patients with anastomotic leakage. Thirty day mortality was 5% in the leakage group compared to 0.6% in the none leakage group (*p* 0.015). Similarly, the 90-day mortality was higher, 8.3 vs. 2% (*p* 0.004). All five patients who died within 90 days in the leakage group had a grade C leakage and required surgery, two men and three women, all with colorectal cancer and severe comorbidity. See Table 2 for mortality in relationship to diagnostic method.

## Discussion

The rate of anastomotic leakage varies in studies, but almost always, the incidence is higher in the rectal resections, and our study confirms that. Overall, the leakage rate was 10% but in rectal resections 18.8%. In procedures with stapled anastomosis and defunctioning stomas, the leakage rates were high, but both were strongly correlated to rectal resections; thus, no conclusions regarding stapled anastomoses can be drawn from this study.

We found that almost one fourth of all CT scans were negative in patients who later were diagnosed with anastomotic leakage. The low sensitivity of these often-used diagnostic methods has been confirmed in other studies [28, 38, 39]. When the CT scan was positive for leakage, it took 8.5 days in mean before leakage was confirmed, compared to 4.3 days in patients who were diagnosed during a reoperation. This may be due to a more ill patient in the surgical group, but it may also illustrate that a negative CT scan seems to mislead the treating physician. This raises the question if we use CT scan too often in early leakages, maybe a second look in the operating theater would be preferable. Maybe postoperative surveillance scores and protocol for leakage diagnosis should be used more often such as routine measurement of C-reactive protein or procalcitonin [40–42]. In previous studies, a question has been raised if there are two different kinds of leakages, one early and another type that present itself later. This is somewhat confirmed in our cohort as 20% (12/60) of our patients had their leakage diagnosed after discharge and at a readmission (1 leakage in grade A, 2 in grade B, and 9 in grade C). This can of course also be influenced by the increased use of enhanced recovery programs discharging patients early [43].

Most patients with a leakage had many other postoperative complications and underwent surgery. More than three fourths (76.3%) of the patients had underwent surgery with the anastomosis taken down, is it possible to reduce this number? Sirois-Giguère et al. describe in an observational study on anastomotic leakage in rectal cancer surgery that 16 out of 37 patients (43%) were treated with transanal drainage with comparable results as the abdominal reintervention group [34]. In our leakage group, we had only 2/22 patients with rectal resections treated with transanal drainage, and perhaps it is possible to retain the anastomosis this way; however, the functional results must be evaluated [1]. In a nationwide study on colon cancer surgery, Krarup et al. describes that in grade C leakages, salvage of the anastomosis was possible in 14.6% (*n* = 74) with small defects or intraoperative findings similar to Hinchey I–II [36]. In our cohort, 9/51 (17.6%) of the patient with grade C leakages had salvage of the anastomosis, and this is somewhat higher than in the Krarup study. The fact that mortality was higher in patients with an anastomotic leakage is not new [44]. However, most patients that died due to anastomotic leakage had severe comorbidity and a malignant disease. All these confirm that anastomotic leakage has major effect on the patients’ life, morbidity, and mortality.

The unselected population with both malign and benign surgery is the strength of our study. We studied the charts in detail, and that is an advantage compared to a registry-based study where details to this extent are difficult to retrieve. However, a retrospective study has limitations in that the data is already existing; the patients’ medical records cannot be redesigned nor can missed information be recreated. However, one of the strengths with a retrospective study is that neither surgeons nor patients know that they are subjects of research. This study includes consecutive patients and is limited to one hospital, and the results are therefore representative for this specific geographic region.

### Conclusion

This study demonstrated that one fourth of all the CT scans that were executed were initially negative for leakage, possibly delaying the diagnosis. Most patients with a grade C leakage will not have an intact anastomosis. An anastomotic leakage leads to significantly more severe postoperative complications, higher rate of reoperations, and higher mortality. An earlier relaparotomy instead of a CT scan and improved postoperative surveillance could possibly reduce the consequences of the anastomotic leakage.
